# Impact of age and sex on neuroinflammation following SARS-CoV-2 infection in a murine model

**DOI:** 10.3389/fmicb.2024.1404312

**Published:** 2024-07-15

**Authors:** Venkatramana D. Krishna, Allison Chang, Holly Korthas, Susanna R. Var, Davis M. Seelig, Walter C. Low, Ling Li, Maxim C. -J. Cheeran

**Affiliations:** ^1^Department of Veterinary Population Medicine, College of Veterinary Medicine, University of Minnesota, Saint Paul, MN, United States; ^2^Graduate Program in Neuroscience, University of Minnesota, Minneapolis, MN, United States; ^3^Department of Experimental and Clinical Pharmacology, College of Pharmacy, University of Minnesota, Minneapolis, MN, United States; ^4^Department of Neurosurgery, University of Minnesota Medical School, Minneapolis, MN, United States; ^5^Comparative Pathology Shared Resource, Masonic Cancer Center, University of Minnesota, Minneapolis, MN, United States

**Keywords:** SARS-CoV-2, COVID-19, brain, neuroinflammation, transcriptomics, mouse model

## Abstract

Severe Acute Respiratory Syndrome Coronavirus 2 (SARS-CoV-2), the etiological agent of COVID-19, is known to infect people of all ages and both sexes. Senior populations have the greatest risk of severe COVID-19, and sexual dimorphism in clinical outcomes has been reported. Neurological symptoms are widely observed in COVID-19 patients, with many survivors exhibiting persistent neurological and cognitive impairment. The present study aims to investigate the impact of age and sex on the neuroinflammatory response to SARS-CoV-2 infection using a mouse model. Wild-type C57BL/6J mice were intranasally inoculated with SARS-CoV-2 lineage B.1.351, a variant known to infect mice. Older male mice exhibited a significantly greater weight loss and higher viral loads in the lung at 3 days post infection. Notably, no viral RNA was detected in the brains of infected mice. Nevertheless, expression of IL-6, TNF-α, and CCL-2 in the lung and brain increased with viral infection. RNA-seq transcriptomic analysis of brains showed that SARS-CoV-2 infection caused significant changes in gene expression profiles, implicating innate immunity, defense response to virus, and cerebrovascular and neuronal functions. These findings demonstrate that SARS-CoV-2 infection triggers a neuroinflammatory response, despite the lack of detectable virus in the brain. Aberrant activation of innate immune response, disruption of blood-brain barrier and endothelial cell integrity, and suppression of neuronal activity and axonogenesis underlie the impact of SARS-CoV-2 infection on the brain. Understanding the role of these affected pathways in SARS-CoV-2 pathogenesis helps identify appropriate points of therapeutic interventions to alleviate neurological dysfunction observed during COVID-19.

## 1 Introduction

Severe acute respiratory syndrome coronavirus 2 (SARS-CoV-2) is the causative agent of the coronavirus disease 2019 (COVID-19) pandemic. COVID-19 symptoms range from mild flu-like illness, fever, fatigue, dry cough and dyspnea to fatal pneumonia and acute respiratory distress (Huang et al., 2020). Since the beginning of the pandemic in March 2020, there have been more than 775 million confirmed cases of COVID-19, with almost 7 million reported deaths (WHO, 2024). SARS-CoV-2 is an enveloped, single-stranded, positive sense RNA virus that belongs to the *Betacoronavirus* genus in the *Coronaviridae* family (Lu et al., 2020; Wu et al., 2020). SARS-CoV-2 consists of four structural proteins: spike (S), membrane (M), envelope (E), and nucleocapsid (N). The S protein mediates viral entry into host cells by binding to the surface receptor angiotensin-converting enzyme 2 (ACE2) (Hoffmann et al., 2020).

Although SARS-CoV-2 primarily infects cells in the respiratory tract, it affects multiple organ systems, including the central nervous system (CNS) (Helms et al., 2020; Puelles et al., 2020; Xydakis et al., 2020). About 10–60% of patients (depending on cohorts studied), infected with SARS-CoV-2, experience a variety of post-acute sequela 1–3 months after the initial infection (Theoharides, 2020; Soriano et al., 2022; Davis et al., 2023; Hastie et al., 2023; Thaweethai et al., 2023; Makhluf et al., 2024). This long-term manifestation of COVID is defined by the World Health Organization (WHO) as “long COVID” when it lasts for at least 2 months with no other attributable causes for the clinical signs (WHO, 2022). A conservative estimate suggests that 10% (>65 million) of SARS-CoV2 infected individuals currently live with long COVID in the world (Ballering et al., 2022).

Long COVID is characterized by numerous symptoms, including persistent fatigue and neurological issues, which may last for many months post-acute SARS-CoV-2 infection (Davis et al., 2021, 2023; Lippi et al., 2023; Thaweethai et al., 2023). In fact, 20–45% of individuals infected with SARS-CoV-2 experience an array of neurological and neurodegenerative issues and cognitive deficits (Patel et al., 2022; O’Mahoney et al., 2023). It has been hypothesized that the presence of viral antigens and chronic inflammation, including the activation of brain mast cells, microglia, astrocytes, and other immune cells, may influence the pathogenesis and duration of long COVID (Hafezi et al., 2021; Theoharides and Kempuraj, 2023). In addition, several lines of evidence indicate that SARS-CoV-2 infection disrupts the blood-brain barrier (BBB) and compromised neurovascular function (Buzhdygan et al., 2020; Raghavan et al., 2021; Reynolds and Mahajan, 2021; Yang et al., 2022; Leng et al., 2023), contributing to neuroinflammation and cognitive deficits.

Animal models play a crucial role in the study of COVID-19 pathogenesis. Wild type laboratory mice are not susceptible to initial lineage A variants of SARS-CoV-2 due to inefficient interaction between the S protein and mouse ACE2 receptor (Ren et al., 2021). To overcome this limitation, various mouse models expressing human ACE2 (hACE2) have been utilized, including K18-hACE2 transgenic mice expressing hACE2 under the control of the cytokeratin 18 promoter (McCray et al., 2007; Yinda et al., 2021), humanized ACE2 mice by replacing endogenous mouse ACE2 with hACE2 using CRISPR/Cas9 knock-in technology (Sun S. H. et al., 2020), Ad5-hACE2 transduced mice (Sun J. et al., 2020), and AAV-hACE2 transduced mice (Israelow et al., 2020). Mouse-adapted SARS-CoV-2 strains have also been developed by multiple laboratories enabling infection of wild type laboratory mice (Dinnon et al., 2020; Gu et al., 2020; Leist et al., 2020; Yan et al., 2022). Further characterization of mouse-adapted SARS-CoV-2 through sequencing showed that the N501Y mutation in the receptor binding domain (RBD) of S protein increased virulence in mice (Gu et al., 2020). Studies found that some of naturally occurring SARS-CoV-2 variants, such as B.1.1.7, B.1.351, and P.1, also possess the N501Y S protein mutation and can efficiently infect wild type laboratory mice (Montagutelli et al., 2021; Niu et al., 2021; Pan et al., 2021; Shuai et al., 2021; Yasui et al., 2022; Zhang et al., 2022).

Clinical studies of patients with COVID-19 have shown that increased age is associated with severe outcomes and higher mortality (Heald-Sargent et al., 2020; Kang and Jung, 2020; Liu et al., 2020; O’Driscoll et al., 2021). Hospital admissions and mortality rates were higher in patients over 65 years old (O’Driscoll et al., 2021). In addition, epidemiological and clinical studies indicate that COVID-19 severity and mortality rates were higher in men than in women (Chen et al., 2020; Guan et al., 2020; Scully et al., 2020; Pivonello et al., 2021). The overarching goal of this study is to assess the effects of age and sex on SARS-CoV-2 infection. We hypothesize that increase in age is associated with enhanced neuroinflammation in a sex-dependent manner following SARS-CoV-2 infection, which impacts the severity of neurological outcomes in COVID-19. To test this hypothesis, the B.1.351 variant of SARS-CoV-2 was used to infect wild type C57BL/6J mice (male and female) at different ages to investigate the neuroinvasive potential of SARS-CoV-2 and associated neuroinflammation.

## 2 Materials and methods

### 2.1 Cells and virus

Vero E6 cells (ATCC CRL-1586, Manassas, VA, USA) were grown in Dulbecco’s modified Eagle medium (DMEM) supplemented with 5% heat inactivated fetal bovine serum (FBS). SARS-CoV-2, isolate hCoV-19/South Africa/KRISP-EC-K005321/2020, NR-54008, lineage B.1.351 was obtained from BEI Resources, NIAID, NIH (Manassas, VA, USA) and propagated in Vero E6 cells. Authenticity of the viral strain was validated by genome sequencing and compared to published sequence (GISAID Accession ID EPI_ISL_678597). Virus titers were determined by focus-forming assay on Vero E6 cells. All procedures with infectious SARS-CoV-2 were performed in certified biosafety level 3 (BSL3) facilities at the University of Minnesota (UMN) using appropriate standard operating procedures (SOPs) and protective equipment approved by the UMN Institutional Biosafety Committee.

### 2.2 Mice

C57BL/6J mice (The Jackson laboratory Stock No. 000664) and K18-hACE2 mice (The Jackson laboratory, Stock No. 034860) were propagated at the UMN and maintained in specific pathogen-free conditions. Mice were housed in groups of 3 to 5 per cage and maintained on a 12 h light/12 h dark cycle with access to water and standard chow diet *ad libitum*. Both male and female C57BL/6J mice at different ages (4-, 10-, and 16-months) or 4-month-old hemizygous K18-hACE2 mice were used in this study.

### 2.3 Infection of mice with SARS-CoV-2

Mice were randomly assigned to infected and uninfected groups with equal numbers of male and female mice in each group. Mice in the infected group were anesthetized with 3% isoflurane/1.5 L/min oxygen in an induction chamber and inoculated intranasally with 1 × 10^5^ focus forming unit (FFU) of SARS-CoV-2 in a volume of 50 μL DMEM, split equally between each nostril. Mice were monitored, and their body weight measured daily for the duration of the experiment. At 3- and 7-days post infection (dpi), mice were euthanized, and lungs and brains were harvested for downstream analysis.

### 2.4 Viral RNA quantification by PCR

Approximately half of the lung and the left hemisphere of each brain were weighed and homogenized in 1 mL of DMEM supplemented with 2% FBS in GentleMACS™ M tube using GentleMACS™ Dissociator (Miltenyi Biotec) with RNA 2.01 program setting. Tissue homogenates were stored in aliquots at −80°C until use. Total RNA was extracted from 250 μL of tissue homogenate using Trizol™ LS reagent (Thermo Fisher scientific, Waltham, MA) according to the manufacturer’s instructions. The purity of RNA was assessed by calculating the ratio of OD_260_/OD_280_. 1 μg of total RNA was reverse transcribed using High-Capacity cDNA reverse transcription kit (Thermo Fisher scientific, Waltham, MA) according to manufacturer’s instructions. RT-qPCR was performed using Fast SYBR Green master mix (Thermo Fisher scientific, Waltham, MA) in a 7500 Fast Real-time PCR system (Applied Biosystems) using the Centers for Disease Control and Prevention RT-PCR primer set targeting SARS-CoV-2 N gene, forward primer 5′-GACCCCAAAATCAGCGAAAT-3′ (2019-nCoV_N1-F), reverse primer 5′-TCTGGTTACTGCCAGTTGAATCTG-3′ (2019-nCoV_N1-R). The following reaction conditions were used: 95°C for 3 min followed by 40 cycles of 95°C for 10 s, 65.5°C for 30 s, and 72°C for 30 s. A standard curve was generated to determine genome copy numbers in the tissue sample by performing a RT-qPCR using synthetic SARS-CoV-2 RNA control (Twist Bioscience, Cat #104043). Viral loads are presented as copies per μg of total RNA.

### 2.5 SARS-CoV-2 focus forming assay

Vero E6 cells were seeded in 24-well tissue culture plates at a density of 1 × 10^5^ cells/well and incubated until the monolayer was 90–100% confluent. Four replicate wells of Vero E6 cells were infected with 100 μL of 10-fold serial dilution of virus or lung/brain homogenate and incubated for 1h with intermittent mixing at 37°C/ 5% CO_2_. After 1 h, 500 μL of overlay medium containing 1.6% microcrystalline cellulose, 2% heat-inactivated FBS in DMEM was added to each well and incubated for 48 h at 37°C/ 5% CO_2_. The cells were fixed with 4% paraformaldehyde for 30 min at room temperature. Fixed cells were washed with PBS containing 0.3% Triton X-100 (PBST), blocked with blocking buffer (1% bovine serum albumin (BSA) and 1% normal goat serum in PBST) for 1 h at room temperature, and then treated with rabbit anti- SARS-CoV-2 nucleocapsid antibody (Sino Biological; 1:2000 dilution) overnight at 4°C. After washing twice with PBST, cells were treated with alkaline phosphatase-conjugated goat anti-rabbit IgG (Thermo Fisher scientific, Waltham, MA; 1:1000 dilution) at room temperature for 1 h. Cells were washed twice with PBST and incubated for 20 min at room temperature in the dark with 1-Step™ NBT/BCIP substrate solution (Thermo Fisher scientific, Waltham, MA). After incubation, wells were washed with deionized water and the numbers of foci were counted. Infectious virus titer in tissue homogenate was expressed as focus forming units (FFU) per g tissue.

### 2.6 Histopathology

Lung samples were immersion-fixed with 4% paraformaldehyde (PFA) for 48 h and washed 3 times with PBS. Samples were routinely processed and embedded in paraffin. From each paraffin block, which contained representative tissue sections from 3 animals, 5 micron sections were cut and stained with hematoxylin and eosin (H&E). Each section was evaluated by a board-certified veterinary pathologist to provide a semi-quantitative assessment of lung injury and a descriptive narrative of lung pathology. The lung injury parameters (perivascular inflammation, interstitial inflammation, interstitial / perivascular edema, alveolar edema, thrombosis, and hemorrhage) were scored (in 0.5 increments) on a 5-point injury scale of increasing severity and extent of tissue involvement. Inflammation was further characterized according to the nature of the cellular infiltrate (i.e., neutrophilic, eosinophilic, mononuclear, histiocytic, or mixed).

### 2.7 Immunohistochemistry

Mouse brain hemispheres were immersion-fixed with 4% PFA for 48 h and washed 3 times with 1X PBS. Brains were embedded in Tissue-Tek OCT (Andwin Scientific, IL) on dry ice. Frozen blocks were stored at −80°C until sectioning. Embedded brains were cut into sagittal sections at 10 μm thickness using a Cryostat (Leica Biosystems Inc). Sections were washed with wash buffer (0.3% Triton-X 100 in 1X PBS) and blocked with blocking buffer (1% normal goat serum, 0.3% Triton-X 100 in 1X PBS) for 60 min at room temperature. Tissue sections were incubated overnight at 4°C with primary antibody against the SARS-CoV-2 nucleocapsid protein (1:1000, Sino Biologicals US Inc, Wayne, PA). After washing 3 times with wash buffer, sections were incubated with goat anti-rabbit IgG-Alexa Fluor™ 594 (1:1000, Thermo Fisher Scientific, Waltham, MA) for 60 min at room temperature. DAPI (4′,6-diamidino-2-phenylindole, Thermo Fisher Scientific, Waltham, MA) was used to label nuclei. Images were captured using Leica DMi8 inverted microscope and Leica LAS software.

### 2.8 RNA-seq analysis

RNA was extracted from homogenized tissue as described above. Isolated RNA samples were submitted to the UMN Genomics Center core facility for library preparation and sequencing as previously described (Jeong et al., 2021; Qu et al., 2023). Briefly, RNA quantities were determined by RiboGreen RNA Quantification assay (Thermo Fisher Scientific, Waltham, MA) and RNA quality was assessed using the Agilent Bioanalyzer (Agilent Technologies). Library preparation was performed using a TruSeq Stranded mRNA Library Prep Kit (Illumina) according to the manufacturer’s instructions. Sequencing was performed on a NovaSeq 6000 platform (Illumina), generating 20 million 150-bp paired-end reads per sample.

Raw sequencing reads were evaluated and trimmed using Trimmomatic (Bolger et al., 2014). Trimmed reads were mapped to the mouse reference genome GRCm38 using HISAT2.^1^ RNA-seq raw counts for each gene were extracted using FeatureCounts (Liao et al., 2014). Exploratory data analysis using PCA was performed on normalized data for outlier detection. One outlier (uninfected, male) was detected and excluded from subsequent analyses (Supplementary Figure 1). Differential gene expression (DGE) analysis was conducted using DESeq2 (1.38.2) (Love et al., 2014). Default DESeq2 normalization and filtering methods were applied.

Data from both sexes (male and female) were analyzed in pairwise comparisons between infected and uninfected males and females, in addition to pairwise comparisons between infected and uninfected mice using the Wald test (design = ∼ sex + infection). Additionally, to assess potential differences in the infection effect between sexes, we performed analyses using design = ∼ sex + infection + sex:infection. Significance for gene differences between groups was determined using an adjusted *p*-value of <0.05 after Benjamini-Hochberg correction. GO gene enrichment analysis was performed using the gseGO function in the ClusterProfiler package (4.6.0) (Yu et al., 2012). Volcano plots were generated using ggplot2 (3.4.0). Hierarchical cluster analysis of the differentially expressed genes (DEGs) was performed using pheatmap (1.0.12) with default parameters (clustering_distance_cols = euclidean, clustering_method = complete). All DEG and pathway enrichment analyses were performed in R (4.2.1).

#### 2.8.1 Profiling cell type composition

The CIBERSORTx web tool was used to determine the cell type composition from the deconvolution analysis of bulk RNA-seq data^2^ (Newman et al., 2019). Single cell RNA-seq data containing CD45+ cells from mouse cortex and hippocampus (Chen et al., 2023) were downloaded from an online dataset (GSE221856). Cells were clustered and labeled using SingleR (2.4.1) and celldex::MouseRNAseqData as a reference, followed by direct manual curation. Labeled cells were used to construct a single-cell reference matrix using default parameters. Imputed cell fractions were generated using relative mode with default settings and 100 permutations. A two-tailed t-test was used to compare differences in immune cell type proportions between SARS-CoV-2 and control groups. An adjusted *p*-value < 0.05 was deemed significant.

### 2.9 Real-time quantitative polymerase chain reaction (RT-qPCR)

Total RNA was extracted from lung and brain homogenates and cDNA was synthesized from 1 μg total RNA as described above. The cDNA was amplified by RT-qPCR using Fast SYBR Green master mix (Thermo Fisher scientific, Waltham, MA) in a 7500 Fast Real-time PCR system (Applied Biosystems) using gene specific primers (Table 1). The specificity of RT-qPCR was assessed by analyzing the melting curves of PCR products. Ct values were normalized to RPL27 gene, and the fold change was determined by comparing virus-infected mice to uninfected controls using 2^–ΔΔCt^ method (Livak and Schmittgen, 2001).

**TABLE 1:** Sequences of primers used for RT-qPCR.

Primer name		Sequence (5′ → 3′)
RPL27	Forward	GCAAAGCTGTCATCGTGAAGAA
	reverse	CTTGTGGGCATTAGGTGATTGT
IL-6	Forward	AGATAACAAGAAAGACAAAGCCAGAG
	reverse	GCATTGGAAATTGGGGTAGGAAG
TNF-α	Forward	CATCTTCTCAAAATTCGAGTGACAA
	reverse	TGGGAGTAGACAAGGTACAACCC
Ifit1	Forward	CTGAGATGTCACTTCACATGGAA
	reverse	GTGCATCCCCAATGGGTTCT
Ifit2	Forward	AGTACAACGAGTAAGGAGTCACT
	reverse	AGGCCAGTATGTTGCACATGG
Tlr7	Forward	ATGTGGACACGGAAGAGACAA
	reverse	GGTAAGGGTAAGATTGGTGGTG
Lyz2	Forward	ATGGAATGGCTGGCTACTATGG
	reverse	ACCAGTATCGGCTATTGATCTGA
B 2m	Forward	TTCTGGTGCTTGTCTCACTGA
	reverse	CAGTATGTTCGGCTTCCCATTC
Mpeg1	Forward	TCCTGTGTGCCTAGTGGAAAA
	reverse	CAAGCGGTTCATCAAGTAGGAT
Gbp7	Forward	CTGGATGATAATAGCGTGTGCT
	reverse	CAAGACAGGTAGTTTCAGGGC

### 2.10 Statistical analyses

Statistical analyses were performed using Prism version 9.5.1 (GraphPad, La Jolla, CA). A two-way Analysis of Variance (ANOVA) with Sidak’s multiple comparison test was used to determine the significance of the difference in body weight changes. A two-way ANOVA with Tukey’s multiple comparison test was used to analyze viral load and relative gene expression of RT-qPCR data. A p-value of less than 0.05 was considered significant.

## 3 Results

### 3.1 Older male mice exhibited greater loss of body weight following infection with SARS-CoV-2

**FIGURE 1:**
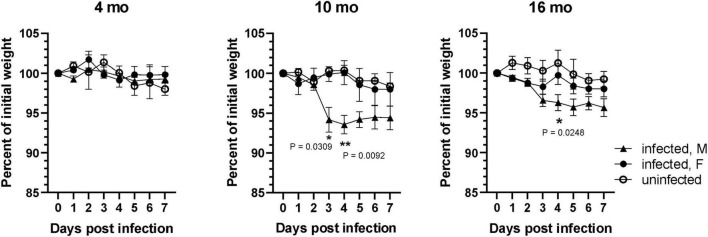
SARS-CoV-2 lineage B.1.135 induced weight loss is higher in older male mice. Male and female C57BL/6J mice at 4, 10, and 16 months of age were infected with 1 × 10^5^ FFU of SARS-CoV-2 lineage B.1.351 via intranasal route. Change in body weight was monitored for 7 days. Error bars indicate standard error of mean (SEM) (*n* = 3 to 4 in each group).

Previous studies have shown that SARS-CoV-2 B.1.351 and other variants with the N501Y mutation in the viral spike protein efficiently infected wild type laboratory mice (Montagutelli et al., 2021; Pan et al., 2021; Shuai et al., 2021; Yasui et al., 2022; Zhang et al., 2022). To determine whether different age groups showed differences in susceptibility to SARS-CoV-2 infection, 4-, 10-, and 16-month-old wild type C57BL/6J mice were infected intranasally with 1 × 10^5^ FFU of SARS-CoV-2 B.1.351 variant (South Africa/KRISP-EC-K005321/20204) and monitored for 7 days, with uninfected age/sex-matched mice as controls. No significant loss of body weight was observed in 4-month-old mice infected with SARS-CoV-2 compared to uninfected mice. However, body weight loss was significantly greater in infected, older male mice at 10- and 16-month of age, than in female mice of the same age (Figure 1). Loss of body weight peaked at 4 dpi in older male mice (male 6.45 ± 1.15% vs female 0 ± 1.49% in 10-month-old; *p* < 0.01 and male 3.7 ± 1.01% vs female 0.25 ± 1.18% in 16-month-old; *p* < 0.05), whereas female mice showed no significant loss in body weight compared to uninfected animals. Further loss in body weight was not observed in male mice after 4 dpi and all mice survived until the experimental end point of 7 dpi.

### 3.2 Older male C57BL/6J mice exhibited prolonged SARS-CoV-2 lung infection compared to younger mice

**FIGURE 2:**
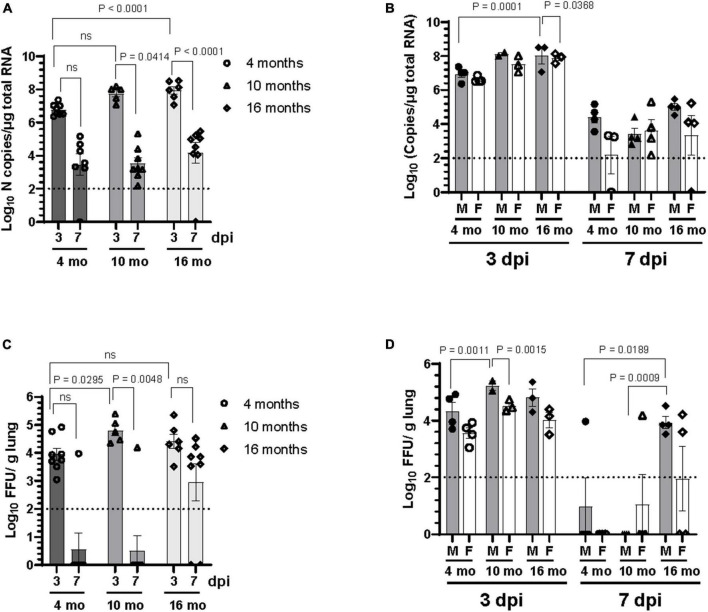
Older male mice are more susceptible to SARS-CoV-2 lung infection. Viral load in the lung homogenate was quantified at 3 dpi and 7 dpi by panel **(A,B)** RT-qPCR for SARS-CoV-2 N gene and expressed as copies of N gene per μg of total RNA, and **(C,D)** by focus forming assay on Vero E6 cells. Error bars indicate standard error of mean (SEM). The dotted line indicates the limit of detection of the assay (*n* = 3 to 4 per group).

The level of viral RNA and infectious virus titer in the lung homogenate of mice were assessed at 3 dpi and 7 dpi. As shown in Figure 2A, viral RNA was detected by RT-qPCR in the lungs of all SARS-CoV-2 infected mice at 3 dpi. Viral RNA was also detectable in most of the lungs of SARS-CoV-2 infected mice at 7 dpi except for one 4-month-old and one 16-month-old mouse. However, the viral RNA load in the lung at 7 dpi was lower compared to 3 dpi. Significantly higher copies of viral RNA were detected at 3 dpi in the lungs of 16-month-old mice compared to 4-month-old mice (Figure 2A), albeit there was no difference in viral RNA levels between different age groups at 7 dpi. Analysis of data based on sex showed that the older male mice at 16 months of age had significantly higher levels of viral RNA in the lung at 3 dpi compared to 4-month-old male mice (Figure 2B). Of note, the viral RNA load in the lungs of female mice was lower than that in male mice at comparable ages at 3 dpi, although the difference did not reach statistical significance except in 16-month-old mice (*p* = 0.036) (Figure 2B). Infectious viral load in the lung was measured by focus forming assay on Vero E6 cells. Although the infectious virus was detected in lungs of SARS-CoV-2 infected mice of all age groups at 3 dpi, infectious virus was infrequent in the lung at 7 dpi (Figure 2C). Additionally, lung virus load was significantly higher at 3 dpi in 10-month-old mice compared to 4-month-old mice. At 7 dpi infectious virus was occasionally detected in the lung of younger mice with 14% of 4-month-old (1 out of 7) and 12.5% of 10-month-old (1 out of 8) mice having detectable virus. In contrast, 75% (6 out of 8) of 16-month-old mice harbored detectable levels of infectious virus in the lung at 7 dpi (Figure 2C), suggesting that the virus clearance is slower in older mice. Analysis of sex-disaggregated data showed that 10-month-old male mice had significantly higher infectious viral load in the lung at 3 dpi compared to 4-month-old male mice. Viral load in the lung of female mice at 3 dpi were lower than male mice in all age groups studied, although the difference was statistically significant only in 10-month-old mice (Figure 2D). At 7 dpi, 25% male (1 out of 4) 4-month-old and 25% female (1 out of 4) 10-month-old mice had detectable virus. While all the 16-month-old male mice had infectious virus in the lung, only 50% (2 out of 4) of female mice showed detectable virus in this age group at 7 dpi (Figure 2D). Collectively, these results indicate that older male mice take more time to clear infectious virus from the lung compared to younger mice.

### 3.3 SARS-CoV-2 infection induced lung inflammation that is most severe in 10-month-old mice with no consistent sex differences

**FIGURE 3:**
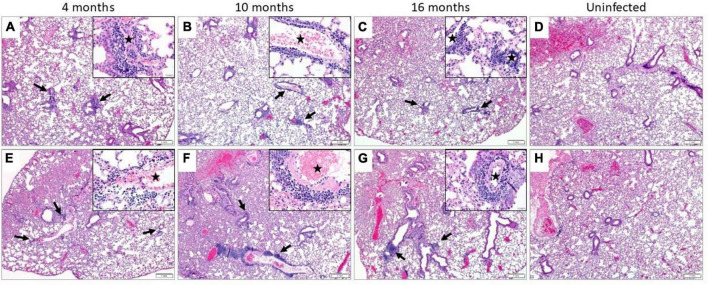
Pulmonary inflammation in SARS-CoV-2 infected mice. Representative images of H&E stained sections. All infected male **(A–C)** and female **(E–G)** mice demonstrated perivascular and interstitial inflammation that was not observed in their uninfected counterparts **(D,H**, respectively). The inflammatory foci were multifocal (black arrows), composed predominantly of lymphocytes and plasma cells and surrounding small and medium-sized vessels (black star, insets) (*n* = 3 males and 3 females in SARS-CoV-2 infected and uninfected group). **(A–H)** = 40X and insets = 400X.

Regardless of age or sex, the lungs from all SARS-CoV-2 infected mice revealed a similar spectrum and, with rare exception, a similar severity of pathology. The infected mice demonstrated multifocal perivascular and interstitial pulmonary inflammation with edema and alveolar histiocytosis (Figure 3 and Supplementary Table 1). In both the male and female mice, the inflammation was most severe in the 10-month-old mice, least severe in the 4-month-old mice, and intermediate in the 16-month-old mice. When considering sex, there was a trend toward more severe inflammation in the female mice compared to male mice at 16 months of age and there was no overall difference between the sexes at 10 and 4 months of age.

### 3.4 SARS-CoV-2 infection induced neuroinflammatory responses at 7 dpi despite lack of detectable virus in the brain

**FIGURE 4:**
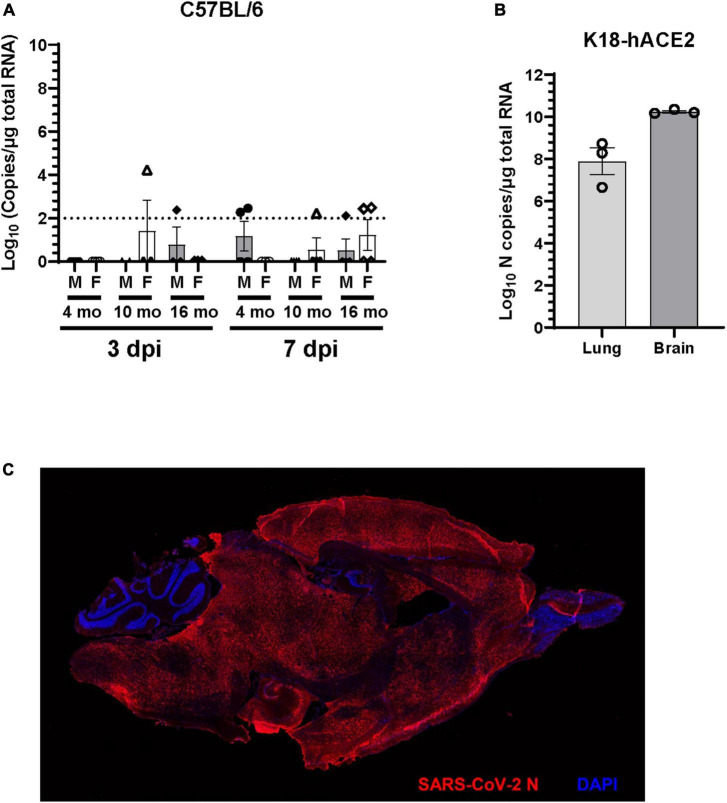
Viral RNA is undetectable in the brain of the majority of C57BL/6J mice. **(A)** Viral RNA in the brain homogenate of C57BL/6J mice infected with 1 × 10^5^ FFU of SARS-CoV-2 was quantified at 3 dpi and 7dpi by RT-qPCR for SARS-CoV-2 N gene and expressed as copies of N gene per μg of total RNA. The dotted line indicates the limit of detection of the assay (*n* = 3 to 4 per group). **(B)** Viral RNA in the lung and brain homogenate of K18-hACE2 mice (*n* = 3) infected with 1 × 10^4^ FFU of SARS-CoV-2 was determined at 7dpi by RT-qPCR for SARS-CoV-2 N gene. **(C)** Representative immunofluorescence image of sagittal brain section of K18-hACE2 mice infected with SARS-CoV-2. Brain sections (*n* = 3 infected and 2 uninfected mice) were immunostained with rabbit polyclonal antibody to SARS-CoV-2 nucleocapsid (N) protein followed by Alexa Fluor 594 conjugated goat anti-rabbit IgG (red). Nucleus were stained with DAPI (blue).

SARS-CoV-2 respiratory infection affects other organ systems, including the central nervous system. To investigate the presence of viral RNA in the brain of C57BL/6J mice infected with SARS-CoV-2 B.1.135 variant, RT-qPCR was performed on total brain RNA at 3 dpi and 7 dpi. Viral RNA was occasionally detected in the brain of SARS-CoV-2 infected mice. At 3 dpi one 10-month-old female (1 out of 3) and one 16-month-old male (1 out of 3) had detectable viral RNA in the brain. At 7 dpi very low viral RNA copies (100–150 copies) were detected in two 4-month-old male (2 out of 4), one 10-month-old female (1 out of 4), one 16-month-old male (1 out of 4), and two 16-month-old female (2 out of 4) mice (Figure 4A). Viral RNA was not detectable in the brains of the majority of mice infected with SARS-CoV-2. Moreover, no infectious virus was detected in the brain of C57BL/6J mice in any of the age groups studied (data not shown). In contrast, we found abundant viral RNA and nucleocapsid protein in brains of 4-month-old K18-hACE2 mice infected with 1 × 10^4^ FFU of SARS-CoV-2 (Figures 4B, C), consistent with previous reports (Jiang et al., 2020; Kumari et al., 2021; Song et al., 2021; Fumagalli et al., 2022).

**FIGURE 5:**
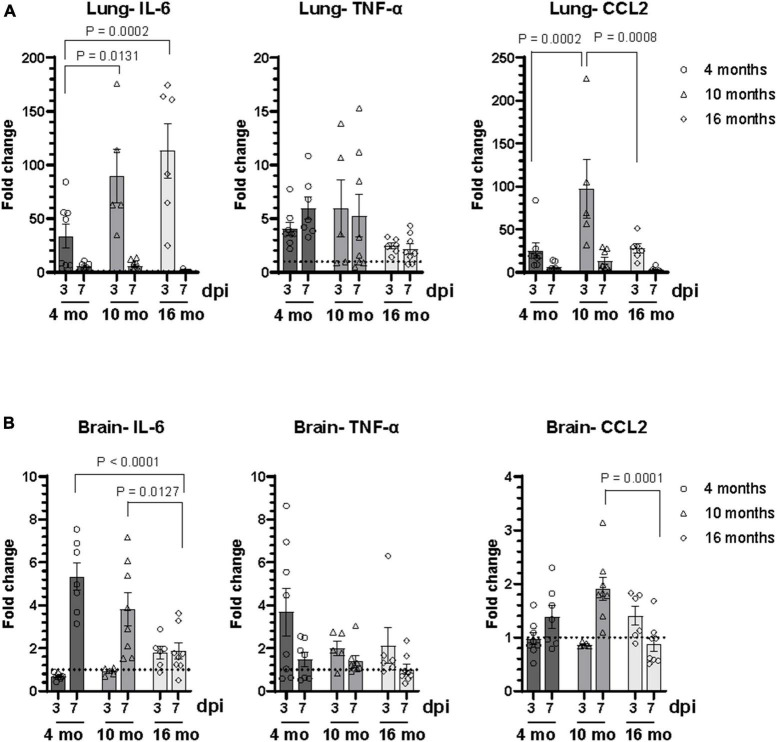
SARS-CoV-2 lineage B.1.135 infection induces an inflammatory response in the brain of C57BL/6J mice despite the absence of virus in the brain at 7 dpi. Relative mRNA expression levels of IL-6, TNF-α, and CCL2 in the **(A)** lung and **(B)** brain of C57BL/6J mice infected with SARS-CoV-2 were analyzed by RT-qPCR. The ΔCT values were normalized to Rpl27 gene expression and represented as fold change (2^ΔΔCT^) over uninfected control. Error bars indicate standard error of mean (SEM). The dotted line represents the mean expression level in uninfected control (*n* = 3 to 4 per group).

Inflammatory cytokine and chemokine mRNA expression levels at 3 dpi and 7 dpi were measured in lungs and brains by RT-qPCR, including IL-6, TNF-α, and CCL2 (Figure 5 and Supplementary Figure 2). As expected, cytokine expression levels in the lung were higher in infected animals at all age groups (Figure 5A). A higher level of IL-6 mRNA expression was observed in lung of older mice compared to 4-month-old mice. While there was no difference in expression of TNF-α at different age groups, 10-month-old mice showed increased CCL-2 expression compared to 4-month and 16-month-old mice (Figure 5A). Analysis of data based on sex revealed differential expression of IL-6 in male and female mice, which was not consistent with age. A significant upregulation of IL-6 mRNA was observed in the lung of 4-month and 10-month-old male mice at both 3 dpi and 7 dpi, compared to age-matched female mice. In contrast, IL-6 expression was significantly higher in the lung of female 16-month-old mice at 3 dpi (Supplementary Figure 2A). Differential expression of TNF-α and CCL2 mRNA in the lung of male and female mice were observed only in 10-month-old mice. Higher level of expression of TNF-α was observed in 10-month-old female mice at both 3 dpi and 7 dpi. CCL2 expression in the lung of 10-month-old mice was higher in males at 3 dpi in contrast to females at 7 dpi (Supplementary Figure 2A).

In the brain at 3 dpi, IL-6 expression was observed only in 16-month-old mice whereas at 7 dpi, IL-6 expression increased in the brain of infected animals of all age groups compared to uninfected age matched controls (Figure 5B). Interestingly, IL-6 expression level in the brain at 7 dpi decreased with increase in age of infected mice. Although there was upregulation of TNF-α expression in the brain of infected mice at 3 dpi compared to uninfected control, there was no difference in the expression levels between different age groups. Higher CCL-2 expression was observed only in the brain of 10-month-old mice (Figure 5B). There was no significant difference in the level of expression of IL-6, TNF-α, and CCL2 in the brain between male and female mice at 7 dpi. However, there was an age dependent decrease in the expression of IL-6 in the brain in male mice at 7 dpi (Supplementary Figure 2B). A significant increase in IL-6 expression in the brain at 3 dpi was observed only in 16-month-old male mice. Additionally, elevated levels of TNF-α in the brain of 4-month-old female mice and CCL2 in 16-month-old male mice were observed at 3 dpi (Supplementary Figure 2B).

### 3.5 SARS-CoV-2 infection induced a widespread innate immune response in the brain

**FIGURE 6:**
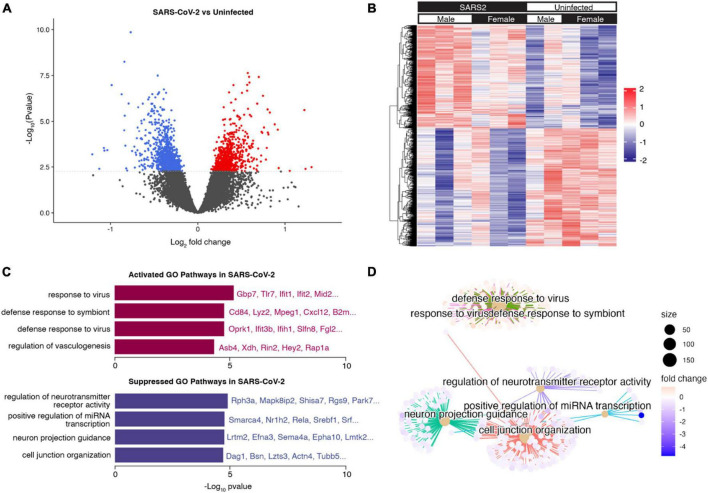
Brain transcriptional response to SARS-CoV-2 infection. **(A)** Volcano plot indicating differentially expressed genes (DEGs) (adjusted *p* < 0.05) in the brains of mice infected with SARS-CoV-2 vs. control. Upregulated DEGs in SARS-CoV-2 infected mice are indicated in red, and downregulated DEGs in SARS-CoV-2 infected mice are indicated in blue. The dotted line corresponds to adjusted *p* = 0.05. **(B)** Hierarchical clustering heatmap view of DEGs. DEGs are presented in the dendrogram along the *Y*-axis. Each column contains expression values for an individual mouse, with groups indicated by the color bars along the *X*-axis. The expression levels from low to high are represented as a color gradient from blue to red, respectively. **(C)** Barplot visualization of the top 8 (by adjusted *p*-value) enriched GO biological process pathways. The significance level is indicated as -Log10 (adjusted *p*-value). **(D)** Cnetplot visualization of the genes in the top 8 (by adjusted *p*-value) enriched GO biological process pathways. Connections between genes and pathway nodes are color coded. The size of each node represents the number of overlapped genes in each GO term and the color represents log-fold change of each gene between groups.

To examine the global effects of SARS-CoV-2 infection on the brain transcriptome, RNA sequencing was performed on brain tissue homogenate collected at 7 dpi from 10-month-old SARS-CoV-2-infected (*n* = 6) and uninfected C57BL/6J mice (*n* = 5). Differential gene expression analysis revealed 1067 differentially expressed genes (DEGs). Of these, 478 genes were significantly upregulated (log FC > 1 and adjusted *p* < 0.05) and 589 genes were significantly downregulated (log FC < 1 and adjusted *p* < 0.05) in SARS-CoV-2-infected mice (Figure 6A). Hierarchical clustering analysis of significant DEGs (adjusted *p*-value < 0.05) revealed distinct transcriptional profiles corresponding with SARS-CoV-2 infection (Figure 6B). Differential gene expression analyses aimed at assessing the influence of sex revealed a minimal number of DEGs, suggesting a limited impact of sex on the overall brain transcriptome.

We performed gene set enrichment analysis (GSEA) to identify the major pathways responsible for the differences between groups (Supplementary Table 2). The top upregulated Gene Ontology (GO) biological process terms in SARS-CoV-2 mice were associated with defense response to virus and other organisms. Among these pathways, common genes included *Tlr7, Ifit1, Ifit2, Ifih1*, and *Gbp7*, all of which play a role in the innate immune response. The top downregulated GO biological process terms in SARS-CoV-2 mice were associated with neuroreceptor activity, axon development, and cell junction organization (Figures 6C, D). These findings provide a foundation for further investigations into the functional implications of these transcriptomic changes in the context of SARS-CoV-2 infection.

To validate the bulk RNA-seq data, selected immune pathway genes that were upregulated in SARS-CoV-2 infected mice compared to controls (Supplementary Figure 3 and Supplementary Table 3) were tested by RT-qPCR. The mRNA expression levels of *Ifit1, Ifit2, Tlr7, Lyz2, B2m, Mpeg1*, and *Gbp7* were evaluated in 4-, 10-, and 16-month-old mice (Supplementary Figure 4). Consistent with the RNA-seq data, RT-qPCR results showed significant upregulation of *Ifit1, Lyz2*, and *Mpeg1* mRNA in the brain of 10-month-old SARS-CoV-2 infected mice compared to uninfected controls. Although, the expression levels of *Ifit2, Tlr7*, and *B2m* were higher than controls, they did not reach statistical significance. There was no change in the expression of *Gbp7* compared to uninfected controls. Further analysis of gene expression data from different age groups of male and female mice revealed that there was age-dependent decrease in expression of *Ifit1*and *Lyz2* genes in male mice (Supplementary Figure 4). While no age dependent change was observed for *Lyz2* expression in female mice, a non-significant trend toward higher *Ifit1* expression levels was observed in older female mice. Interestingly, the expression levels of both *Ifit1* and *Lyz2* were greater in 16-month-old female mice compared to age-matched male mice. *Mpeg1* expression was elevated only in 10-month-old female mice. These results demonstrate that age and sex modify the expression of innate immunity-related genes in the brain in response to SARS-CoV-2 infection.

**FIGURE 7:**
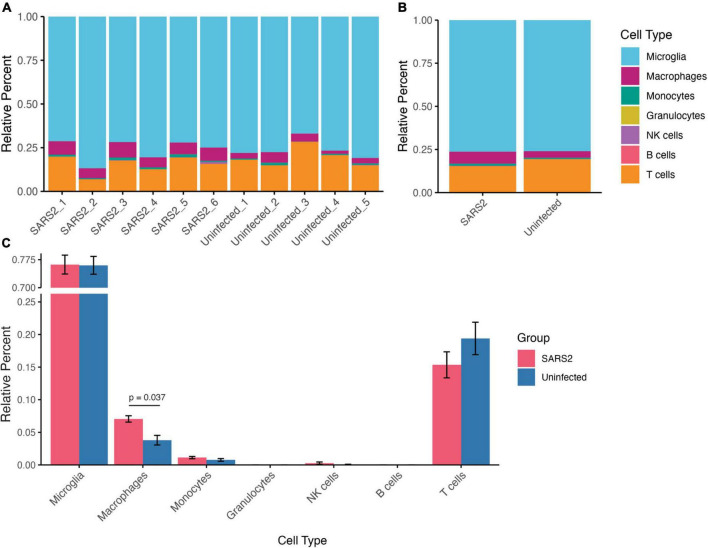
Relative composition of immune cell types in the brain derived from deconvolution analysis of the RNA-seq data using CIBERSORTx. **(A)** Stacked bar plots indicating the cell type proportion in each sample (left), and **(B)** average cell type proportion (right). **(C)** Bar plots comparing immune cell proportions between infected and uninfected mice. Values are means ± SEM.

To dissect specific immune cell subsets from the bulk RNA-seq data, we used CIBERSORTx algorithms to determine immune cell type proportions in each sample (Newman et al., 2019). Among the identified cell types, microglia constituted the majority of cells (SARS-CoV-2 76.2% ± 6.2%, Uninfected 76.0% ± 5.3%), followed by T cells (SARS-CoV-2 15.4% ± 4.9%, Uninfected 19.4% ± 5.6%), macrophages (SARS-CoV-2 7.1% ± 1.2%, Uninfected 3.8% ± 1.7%), monocytes (SARS-CoV-2 1.1% ± 0.43%, Uninfected 0.77% ± 0.46%), and natural killer (NK) cells (SARS-CoV-2 0.28% ± 0.44%, Uninfected 0.050% ± 0.11%) (Figures 7A, B). Intriguingly, the proportion of macrophages was significantly higher in SARS-CoV-2 infected mice compared to uninfected controls (*p* = 0.0372) (Figure 7C). These findings underscore the impact of SARS-CoV-2 infection on immune cell composition within the brain microenvironment, highlighting the special recruitment of macrophages.

### 3.6 SARS-CoV-2 infection induced downregulation of vascular-related pathways in the brain

Among the downregulated pathways, GO enrichment analysis showed that pathways related to cell junction organization and regulation of vasculogenesis were altered in SARS-CoV-2-infected brains. Several genes/proteins involved in maintaining the blood-brain barrier (BBB) integrity were downregulated, including Cldn5, a member of the claudin family. Claudins are key components of tight junctions between adjacent endothelial cells that regulate the permeability of the BBB (Morita et al., 1999; Luissint et al., 2012). Multiple proteins with functions related to regulation of endothelial cell-matrix adhesion or cell-cell junction organization (Ajm and Pard6a, Nectin1, Rcc2, Cbln1) were also expressed at significantly lower levels in infected brains. Consistent with the GO analysis observations, we also found a significant decrease in expression of cadherin proteins (Cdh1, Cdh22, Cdh15, Cdh19) in SARS-CoV-2 infected brains. Cadherins are transmembrane proteins that mediate cell–cell adhesion and cell-cell junction organization (Togashi et al., 2009; Maître and Heisenberg, 2013). These results suggest that SARS-CoV2 infection may lead to disruption of BBB integrity and neurovascular function, in addition to triggering the innate immune response in the brain. Further studies are required to experimentally demonstrate BBB permeability after SARS-CoV-2 infection.

## 4 Discussion

Increased age is considered as one of the major risk factors for severe COVID-19 outcomes (Dudley and Lee, 2020; Heald-Sargent et al., 2020; Kang and Jung, 2020; Liu et al., 2020). Epidemiological studies suggest that patients over 65 years of age account for 80% of COVID-19 hospitalizations with a 20-fold higher mortality rate compared to those under 65 years (O’Driscoll et al., 2021). However, comorbidities such as cardiovascular disease, diabetes, and chronic lung diseases also increase with age and may influence the severity of COVID-19 outcome in older patients. Emerging evidence also indicate that COVID-19 severity and mortality are higher among men than women (Chen et al., 2020; Gebhard et al., 2020; Guan et al., 2020; Scully et al., 2020; Pivonello et al., 2021). In the present study, we investigated the effect of age on SARS-CoV-2 pathogenesis by comparing the response to infection in 4-, 10-, and 16-months old C57BL/6J mice. Using age/sex-disaggregated data from SARS-CoV-2 infected mice, we assessed the impact of age and sex on SARS-CoV-2 pathogenesis. We used a naturally occurring isolate of SARS-CoV-2 beta variant, B.1.135, capable of infecting wild type laboratory mice to induce severe pathological lesions and inflammatory response in the lung (Pan et al., 2021; Shuai et al., 2021; Yasui et al., 2022).

Our results on SARS-CoV-2 infection in a wild-type mouse model reflects the age and sex dependent increase in disease severity reported in humans. We found that the severity of disease outcomes increased in older male mice compared to young or female mice as evidenced by the significant loss of body weight at 3 to 4 dpi, increased viral RNA load in the lung at 3 dpi, and higher percentage of older male mice harboring infectious virus in the lung at 7 dpi. Corroborating our work, earlier studies reported no significant weight loss in 8-week-old young adult C57BL/6 mice infected with the B.1.135 variant, despite the presence of pathological lung lesions and inflammatory responses (Montagutelli et al., 2021; Pan et al., 2021; Shuai et al., 2021). However, Yasui et al. (2022) reported weight loss in infected 8-week-old Balb/c mice or when infected at higher viral dose (1 × 10^6^ pfu) in C57BL/6 mice. These authors also reported increased weight loss and fatality in aged Balb/c mice (Yasui et al., 2022). These reports suggest that in addition to age and sex, the strain/genetic background of mice could be an important component of the SARS-CoV2 murine models.

Neurotropism of SARS-CoV-2 is still under debate. Several *in vitro* studies showed that the cells of the CNS, particularly astrocytes and neurons, support SARS-CoV-2 replication (Bullen et al., 2020; Jacob et al., 2020; Ramani et al., 2020; Wang et al., 2021; Andrews et al., 2022; Crunfli et al., 2022; Roczkowsky et al., 2023). In contrast, viral RNA was either absent (Khan et al., 2021; Lee et al., 2021, 2022; Yang et al., 2021) or present in low levels in a subset of the autopsy brain samples from fatal COVID-19 patients (Kantonen et al., 2020; Matschke et al., 2020; Puelles et al., 2020; Solomon et al., 2020; Meinhardt et al., 2021; Roczkowsky et al., 2023). Consistent with these findings in humans, SARS-CoV-2 RNA was not detected in the brain of majority of infected wild-type mice, with some brains containing very low levels of viral RNA, and no infectious virus was detected in brains of mice in the present study.

COVID-19 is characterized by pronounced inflammation, excess cytokine production, and alterations in both the innate and adaptive immune responses (O’Connell and Aldhamen, 2020). Although the underlying mechanisms contributing to brain pathology are not fully understood, we observed significant changes in the transcriptome of SARS-CoV-2 infected brains that point to the involvement of the innate immune system. DEG and GSEA analysis identified upregulation of several genes that modulate the innate immune response. Notable among them is *Tlr7*, a type of toll-like receptor, which is responsible for activation of innate immune responses and regulation of cytokine production (Jung and Lee, 2021). TLR7 was identified as a cellular detector of ssRNA of SARS-CoV-2, which leads to inflammasome activation and production of pro-inflammatory cytokines and interferons via NF-κB activation (Bender et al., 2020; Khanmohammadi and Rezaei, 2021; Salvi et al., 2021; Manik and Singh, 2022). Recent reports link genetic *Tlr7* deficiencies with more severe COVID-19 infection in young individuals, connecting TLR7 with host resistance and disease outcome (van der Made et al., 2020; Asano et al., 2021; Abolhassani et al., 2022).

Using a mouse-adapted SARS-CoV-2, Beer et al. (2022) showed that the age-dependent increase in disease severity is due to impaired interferon response in older mice. IFIT1 and IFIT2 (also known as ISG56 and ISG54, respectively) are interferon induced proteins primarily known for their roles in antiviral defense by restricting viral replication and modulating immune responses to combat viral infections (Fensterl and Sen, 2015). *Ifit1* and *Ifit2* are induced in response to dsRNA, type I and type II IFNs, and infection by various viruses (Guo et al., 2000). These factors have been shown to inhibit virus replication by binding to eIF3 and limiting translation of viral mRNA (Hui et al., 2005; Terenzi et al., 2006). IFIT1 and IFIT2 exert antiviral activity against the SARS-CoV-2 virus by binding directly to capped viral mRNA to inhibit translation or replication (Mears and Sweeney, 2018). Although we did not assess the level of expression in the lung, *Ifit1* expression was higher in the brain of infected 10-month-old mice compared to controls, implicating IFIT in modulating the host immune response. Furthermore, we found an age-dependent decrease in gene expression of *Ifit1* in the brain of male mice when assessed by RT-qPCR. *Ifit1* expression was lowest in 16-month-old male mice, the group that had maximum viral load in the lung, suggesting impaired *Ifit1* expression in older male mice. Intriguingly, age-dependent reduction in *Ifit1* expression was not observed in female mice.

Furthermore, several genes including *Mpeg1* and *Cd84* are involved in macrophage activation and phagocytosis (Ellett et al., 2011; Orecchioni et al., 2019). Macrophages act as important sentinel cells in peripheral organs where they monitor surrounding tissue for invading pathogens, ingest and kill pathogens, and produce and secrete cytokines/chemokines to regulate the immune response (Kosyreva et al., 2021). Many recent studies highlight the role of macrophages in the pathogenesis of SARS-CoV-2 virus infection in the lungs (Abassi et al., 2020; Kosyreva et al., 2021; Meidaninikjeh et al., 2021; Lian et al., 2022). However, there remains a gap in the understanding of neuroinflammatory responses associated with COVID-19 in the brain. Taken together, our results imply that macrophages might play a crucial role in the progression of SARS-CoV-2 infection in the brain; however, further investigation is required to elucidate the exact factors driving macrophage activation and underlying molecular events governing their phenotype at various stages of infection.

Previous studies have demonstrated that SARS-CoV-2 spike protein can disrupt the function and integrity of the blood–brain barrier (BBB) (DeOre et al., 2021; Wang et al., 2023). Notably, reports of BBB disruption and leakage were documented in 58% of COVID-19 patients across 31 case studies involving individuals with neurological manifestations. In 60% of these patients, abnormal macrophages as well as microglial activation were present in the brain, indicating BBB disruption and leading to expression of proinflammatory molecules (Bellon et al., 2021). Importantly, SARS-CoV-2 induced disruption of the BBB and its link to neuroinflammation are supported by our transcriptomic analysis, which showed the downregulation of pathways related to endothelial cell junction organization and regulation of vasculogenesis, along with the upregulation of innate immune response, in the brain following SARS-CoV-2 infection. Furthermore, deconvolution analysis of our bulk transcriptomic data revealed a higher macrophage proportion in the brains of infected mice compared to uninfected controls, suggesting infiltration of peripheral macrophages into the brain parenchyma through disrupted BBB. These infiltrating macrophages can produce pro-inflammatory cytokines that act on resident microglia, which could contribute to the observed neuroinflammatory response. The increased macrophage proportion aligns with previous reports of macrophage infiltration in the brain of individuals with COVID-19 (Lee et al., 2021, 2022; Schwabenland et al., 2021). Collectively, these findings support the possibility of BBB disruption, subsequent peripheral immune cell infiltration, and activation of the innate immune response in the brain following SARS-CoV-2 infection. These processes likely contribute to neuroinflammation and neuronal cell death, as indicated by the suppression of axonogenesis and neurotransmitter receptor activity pathways.

The results of the present study are consistent with previous *in vitro* experiments and clinical studies that demonstrate that SARS-CoV-2 disrupts the BBB (Buzhdygan et al., 2020; Raghavan et al., 2021; Reynolds and Mahajan, 2021; Yang et al., 2022). We have shown transcriptional *in vivo* evidence that cerebrovascular changes and inflammation occur upon infection, which provide insights into the mechanisms underlying the neurological symptoms of COVID-19. A comprehensive investigation into mechanisms by which SARS-CoV-2 induces BBB disruption and immune dysfunction may offer novel avenues for therapeutic interventions in the management of COVID-19.

## 5 Conclusion

In summary, our findings indicate that SARS-CoV-2 variant B.1.135 infection induces a neuroinflammatory response despite the lack of detectable virus in the brain. Age and sex modify the susceptibility and severity of SARS-CoV-2 infection, highlighting the importance of considering these factors in the context of COVID-19 research. An activated innate immune response, compromised BBB integrity, and suppressed neuronal activities underlie the pathogenic impact of SARS-CoV-2 infection on the brain and is indicative of indirect mechanisms that impact CNS outcomes despite the absence of virus replication in the brain. Further studies are required to determine the long-term neuropathological changes due to SARS-CoV2 infection and to elucidate the underlying mechanisms that drive the neurological manifestations of COVID-19.

## Data availability statement

The datasets presented in this study can be found in online repositories. The names of the repository/repositories and accession number(s) can be found here: https://www.ncbi.nlm.nih.gov/geo/, accession number: GSE237092.

## Ethics statement

The animal study was approved by the University of Minnesota Institutional Animal Care and Use Committee. The study was conducted in accordance with the Federal/State legislation and institutional requirements.

## Author contributions

VK: Investigation, Methodology, Data curation, Formal analysis, Visualization, Writing – original draft. AC: Methodology, Data curation, Formal analysis, Visualization, Writing – original draft. HK: Methodology, Writing – original draft. SV: Methodology, Writing – original draft. DS: Methodology, Formal analysis, Visualization, Writing – original draft. LL: Conceptualization, Funding acquisition, Supervision, Methodology, Writing – review and editing. WL: Conceptualization, Funding acquisition, Supervision, Methodology, Writing – review and editing. MC: Conceptualization, Funding acquisition, Supervision, Methodology, Writing – review and editing.

## Footnotes

1
https://doi.org/10.1038/nmeth.3317

2
https://cibersortx.stanford.edu
