# Supplementary material for: Impact of age and sex on neuroinflammation following SARS-CoV-2 infection in a murine model
Source: Front Microbiol. 2024 Jul 15;15:1404312. doi: 10.3389/fmicb.2024.1404312 (PMC11284165; doi:10.3389/fmicb.2024.1404312)
Supplement: Supplementary file 1 [file Data_Sheet_1.docx]

Supplementary Material

# Supplementary Figures and Tables

## Supplementary Figures


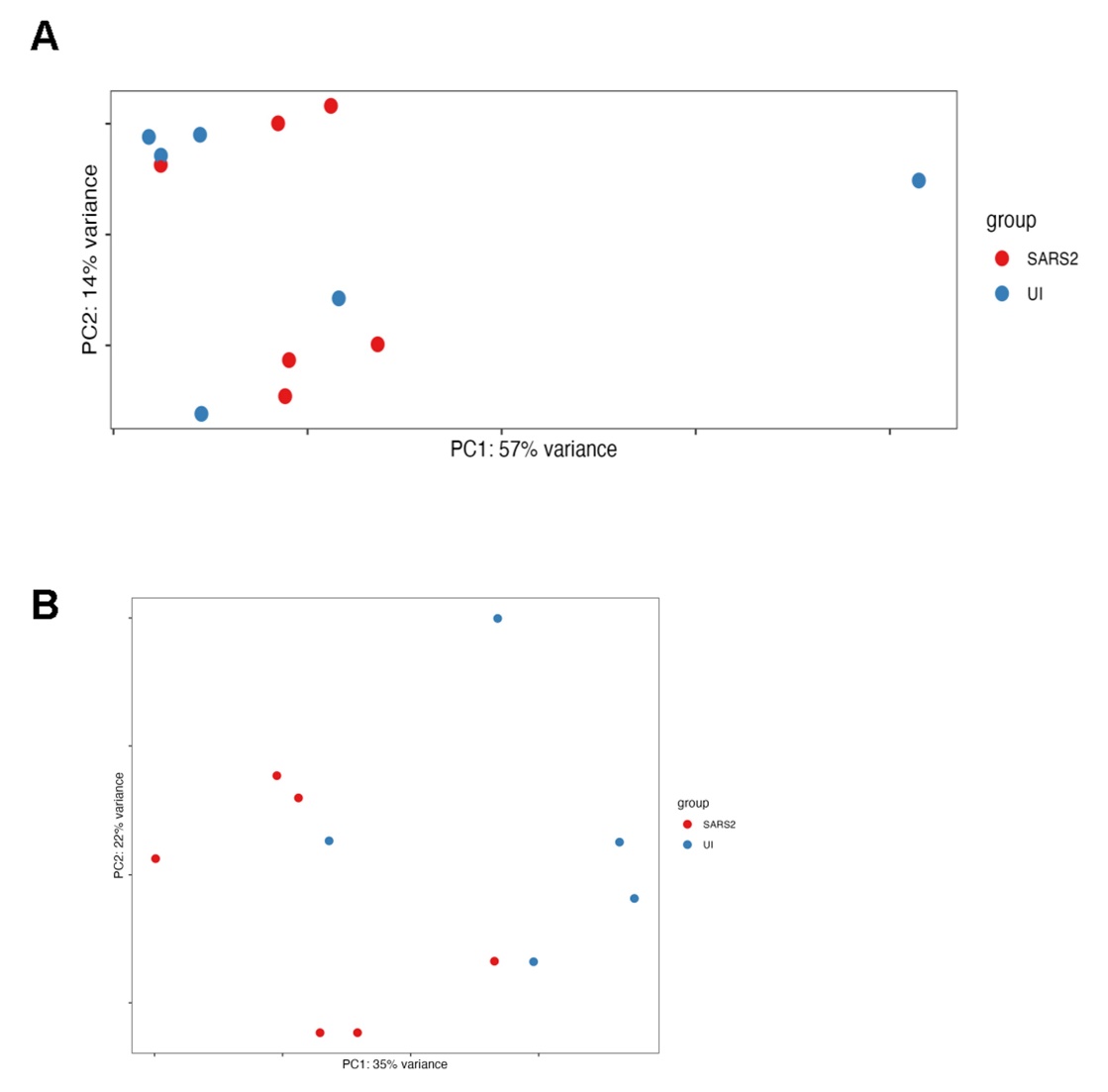


**Supplementary Figure S1.** PCA plots of the SARS-CoV-2 infected and Uninfected groups (A) before and (B) after outlier removal.


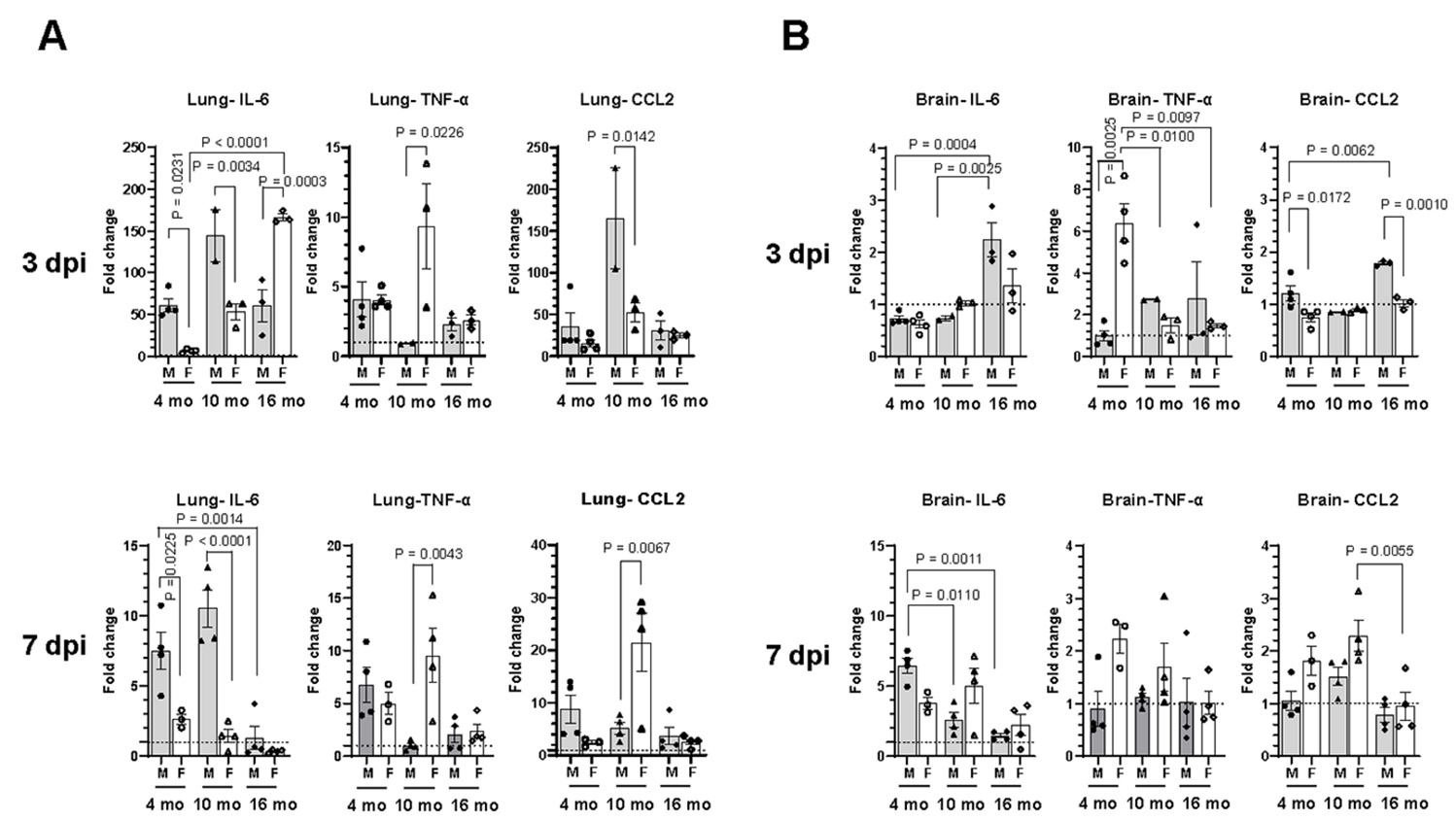


**Supplementary Figure S2.** **SARS-CoV-2 lineage B.1.135 infection induces an inflammatory response in the brain of C57BL/6J mice despite absence of virus in the brain.** Relative mRNA expression levels of IL-6, TNF-α, and CCL2 in the **(A)** lung and **(B)** brain of C57BL/6J mice infected with SARS-CoV-2 were analyzed by RT-qPCR. Error bars indicate standard error of mean (SEM). The dotted line represents the mean expression level in uninfected control. (n=3 to 4 per group).


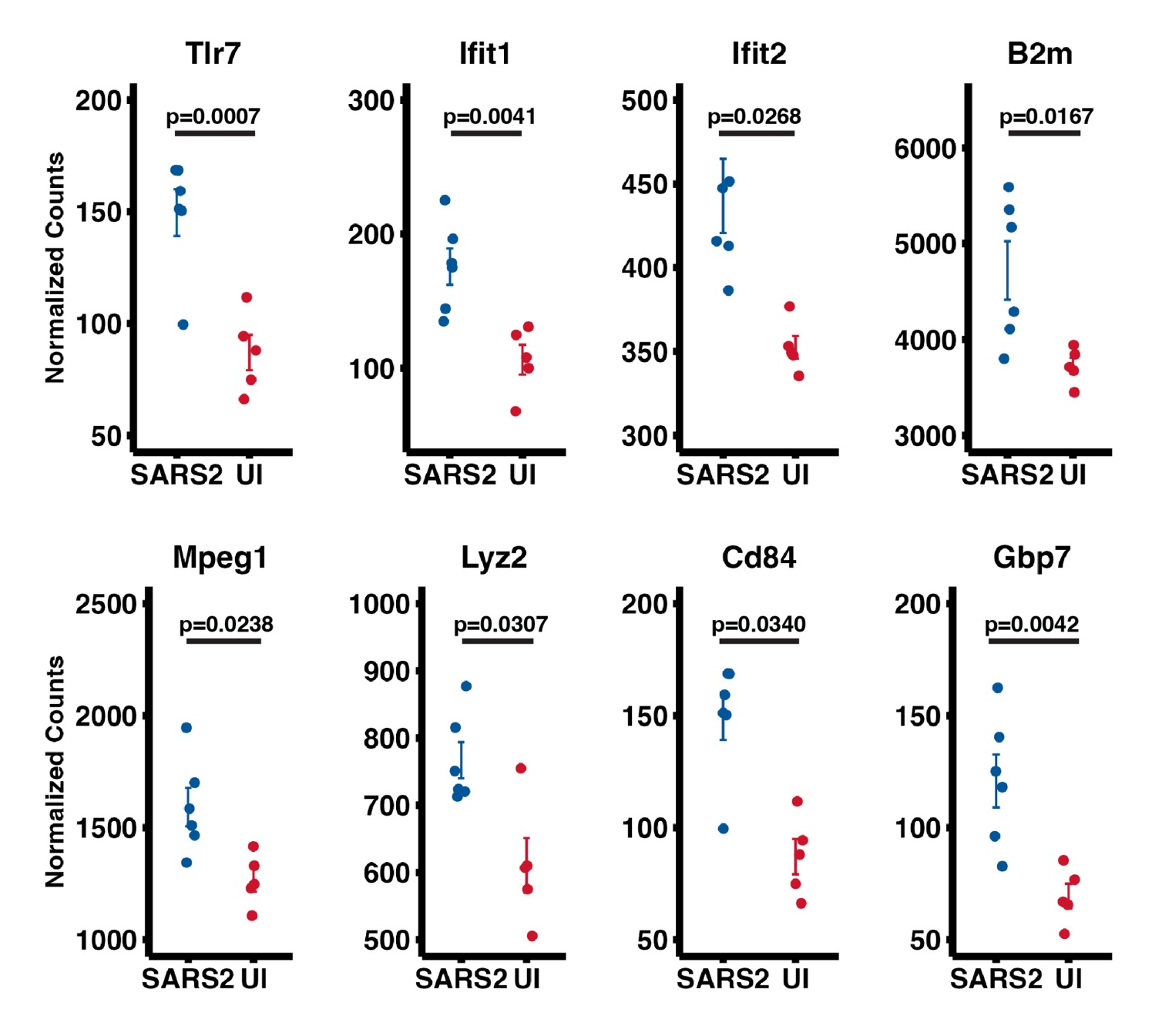


**Supplementary Figure S3.** Plots showing DESeq2 normalized gene expression level of selected DEGs shared among GO immune pathways upregulated in SARS-CoV-2 mice compared to uninfected mice. Benjamini-Hochberg corrected p values are based on DEseq2 analysis. Mean values ± SEMs are shown.


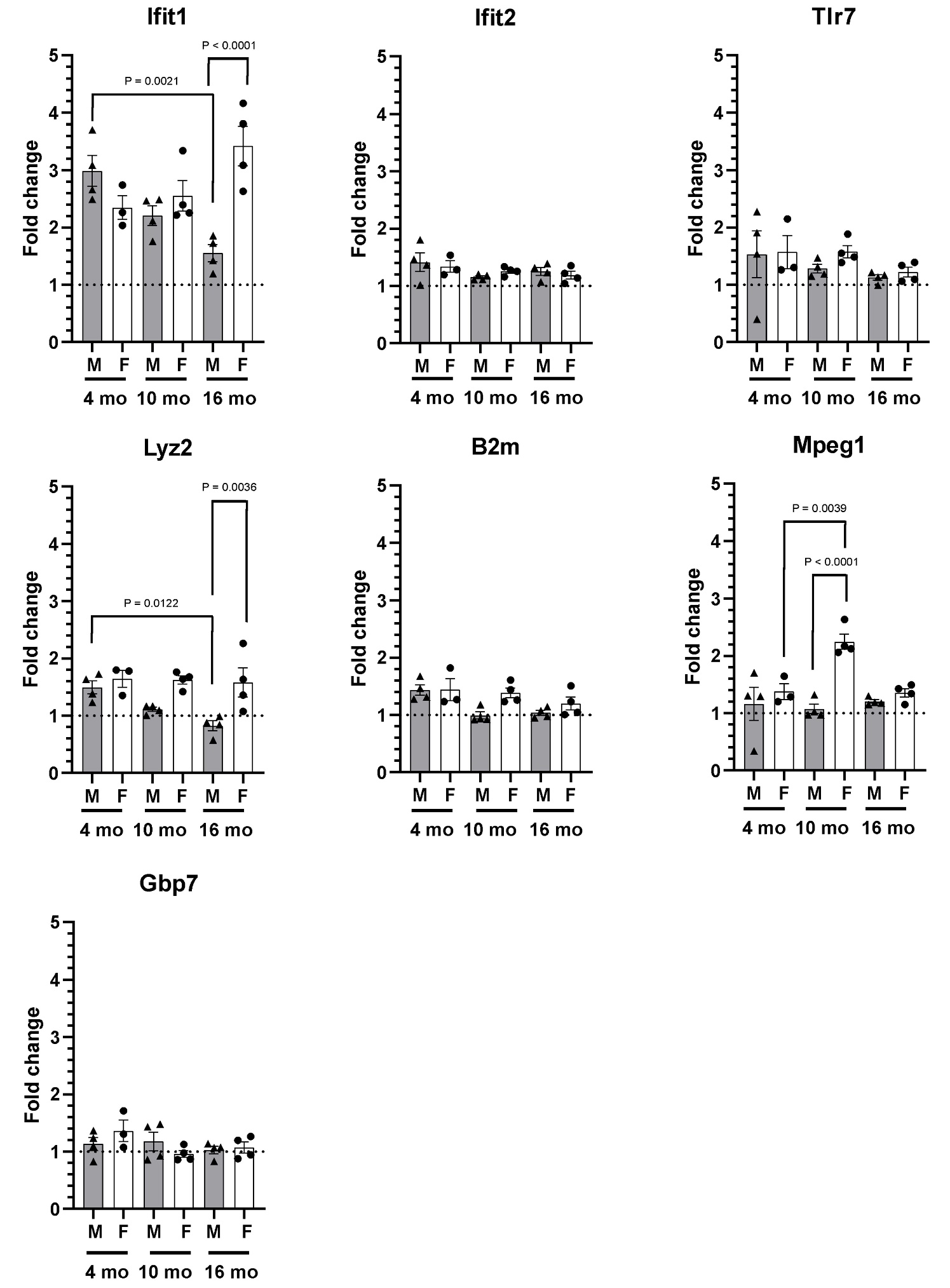


**Supplementary Figure S4. SARS-CoV-2 lineage B.1.135 infection induces an innate immune response in the brain of C57BL/6J mice at 7 dpi.** Relative gene expression levels of Ifit1, Ifit2, Tlr7, Lyz2, B2m, Mpeg1, and Gbp7 were analyzed by RT-qPCR. The ΔCT values were normalized to Rpl27 gene expression and represented as fold change (2^-ΔΔCT^) over uninfected control. The dotted line represents the mean expression level in uninfected control. (n=3 to 4 per group).

## Supplementary Tables

**Supplementary Table S1.** Histopathology scoring of lung sections

| **Animal** | **Group** | **Sex** | **Age (months)** | **Perivascular inflammation** | **Interstitial Inflammation** | **Interstitial Edema** | **Alveolar Edema** | **Thrombosis** | **Hemorrhage** |
| --- | --- | --- | --- | --- | --- | --- | --- | --- | --- |
| 21358 | SARS | M | 4 | 2 | 2 | 1 | 2 | 0 | 2 |
| 21359 | SARS | M | 4 | 2 | 1 | 1 | 1 | 0 | 1 |
| 21360 | SARS | M | 4 | 1 | 2 | 1 | 2 | 9 | 2 |
| 21545 | SARS | F | 4 | 2 | 1 | 1 | 1 | 0 | 1 |
| 21365 | SARS | F | 4 | 2 | 2 | 1 | 1 | 0 | 1 |
| 21318 | SARS | F | 4 | 2 | 2 | 1 | 1 | 0 | 1 |
| 21905 | Uninfected | M | 4 | 1 | 1 | 2 | 1 | 0 | 2 |
| 21906 | Uninfected | M | 4 | 1 | 1 | 2 | 1 | 0 | 2 |
| 21907 | Uninfected | M | 4 | 0.5 | 2 | 2 | 1 | 0 | 1 |
| 21477 | Uninfected | F | 4 | 0.5 | 1 | 1 | 1 | 0 | 2 |
| 21543 | Uninfected | F | 4 | 1 | 2 | 1 | 1 | 0 | 1 |
| 21909 | Uninfected | F | 4 | 2 | 2 | 2 | 1 | 0 | 1 |
| 21192 | SARS | M | 10 | 3 | 2 | 2 | 1 | 0 | 1 |
| 21193 | SARS | M | 10 | 3 | 1 | 1 | 1 | 0 | 1 |
| 21260 | SARS | M | 10 | 3 | 1.5 | 1 | 1 | 0 | 1 |
| 21195 | SARS | F | 10 | 1 | 2 | 2 | 2 | 0 | 1 |
| 21212 | SARS | F | 10 | 4 | 2 | 2 | 1 | 0 | 1 |
| 21252 | SARS | F | 10 | 3 | 2 | 2 | 1 | 0 | 2 |
| 21209 | Uninfected | M | 10 | 1 | 1 | 1 | 2 | 0 | 1 |
| 21646 | Uninfected | M | 10 | 1 | 1 | 1 | 2 | 0 | 1 |
| 21648 | Uninfected | M | 10 | 2 | 1 | 1 | 2 | 0 | 1 |
| 21226 | Uninfected | F | 10 | 1 | 1 | 2 | 1 | 0 | 1 |
| 21649 | Uninfected | F | 10 | 1 | 1 | 2 | 1 | 0 | 1 |
| 2150 | Uninfected | F | 10 | 1 | 1 | 2 | 1 | 0 | 1 |
| 20894 | SARS | M | 16 | 2 | 1 | 1 | 2 | o | 2 |
| 20896 | SARS | M | 16 | 3 | 1 | 1 | 2 | 0 | 1 |
| 21034 | SARS | M | 16 | 2 | 1 | 2 | 1 | 0 | 1 |
| 20842 | SARS | F | 16 | 3 | 2 | 2 | 1 | 0 | 2 |
| 20897 | SARS | F | 16 | 2 | 1 | 1 | 1 | 0 | 2 |
| 21038 | SARS | F | 16 | 3 | 1 | 1 | 1 | 0 | 2 |
| 21222 | Uninfected | M | 16 | 1 | 1 | 1 | 1 | 0 | 1 |
| 21343 | Uninfected | F | 16 | 1` | 0.5 | 1 | 1 | 0 | 1 |

Histology scorings: 0 – Not observed; 1 – Minimal; 2 – Mild; 3 – Moderate; 4 - Marked

**Supplementary Table S2.** Gene Set Enrichment Analysis (GSEA) enriched GO pathways.

| **Pathway** | **Size** | **ES** | **NES** | **pvalue** | **FDR** |
| --- | --- | --- | --- | --- | --- |
| regulation of vasculogenesis | 16 | 0.75 | 2.18 | 0.00 | 0.026 |
| positive regulation of miRNA transcription | 42 | -0.57 | -2.11 | 0.00 | 0.026 |
| bone morphogenesis | 79 | -0.49 | -2.10 | 0.00 | 0.026 |
| neuron projection guidance | 214 | -0.36 | -1.80 | 0.00 | 0.026 |
| defense response to virus | 197 | 0.37 | 1.79 | 0.00 | 0.026 |
| defense response to symbiont | 197 | 0.37 | 1.79 | 0.00 | 0.026 |
| axon guidance | 213 | -0.36 | -1.79 | 0.00 | 0.026 |
| response to virus | 236 | 0.35 | 1.75 | 0.00 | 0.026 |
| cell junction organization | 671 | -0.26 | -1.49 | 0.00 | 0.028 |
| regulation of neurotransmitter receptor activity | 60 | -0.53 | -2.14 | 0.00 | 0.028 |
| miRNA transcription | 56 | -0.53 | -2.09 | 0.00 | 0.028 |
| axon development | 474 | -0.29 | -1.58 | 0.00 | 0.028 |
| axonogenesis | 431 | -0.29 | -1.60 | 0.00 | 0.030 |
| defense response to other organism | 595 | 0.26 | 1.47 | 0.00 | 0.030 |

**Supplementary Table S3.** Cell type expression and function of selected immune-pathway gen

| **Gene Name** | **Cell Type** | **Function** | **References** |
| --- | --- | --- | --- |
| **Tlr7** | Macrophage, Microglia, astrocytes | Innate immune receptor that recognizes single-stranded RNA | (Li et al., 2021; Michaelis et al., 2019) |
| **Ifit1and Ifit2** | Low basal expression, enhanced in response to IFNs and viral infection | Binds to capped RNA to inhibit viral replication and translational initiation | (Mears and Sweeney, 2020; Pidugu et al., 2019; Terenzi et al., 2007) |
| **B2m** | Distinct expression in macrophages and immune cells | Participate in formation of MHC class I complexes, facilitates presentation of viral antigens on infected cells to T cells for immune recognition | (Wang et al., 2022) |
| **Mpeg1** | Most abundant in immune cells | Promotes phagocytosis and antimicrobial activity in macrophages | (Bayly-Jones et al., 2020) |
| **Lyz2** | Myeloblasts, macrophages, and neutrophils | Exhibit antimicrobial activity by catalyzing the hydrolysis of peptidoglycan in bacterial cell walls | (Orthgiess et al., 2016) |
| **Cd84** | Monocytes, macrophages, granulocytes, and dendritic cells | Signaling molecule that regulates immune cell activation and cell-cell interactions | (Cuenca et al., 2019; Sintes et al., 2010) |
| **Gbp7** | B-cells, T-cells, NK-cells, monocytes, granulocytes and dendritic cells | Interferon (IFN)-inducible GTPase that plays important roles in host defense against a range of bacterial, viral and protozoan pathogens | (Steffens et al., 2020) |

**References**

1. Bayly-Jones, C., Pang, S.S., Spicer, B.A., Whisstock, J.C., Dunstone, M.A., 2020. Ancient but Not Forgotten: New Insights Into MPEG1, a Macrophage Perforin-Like Immune Effector. Front. Immunol. 11, 581906. https://doi.org/10.3389/fimmu.2020.581906
2. Cuenca, M., Sintes, J., Lányi, Á., Engel, P., 2019. CD84 cell surface signaling molecule: An emerging biomarker and target for cancer and autoimmune disorders. Clin. Immunol. 204, 43–49. https://doi.org/10.1016/j.clim.2018.10.017
3. Li, L., Acioglu, C., Heary, R.F., Elkabes, S., 2021. Role of astroglial toll-like receptors (TLRs) in central nervous system infections, injury and neurodegenerative diseases. Brain. Behav. Immun. 91, 740–755. https://doi.org/10.1016/j.bbi.2020.10.007
4. Mears, H.V., Sweeney, T.R., 2020. Mouse Ifit1b is a cap1-RNA–binding protein that inhibits mouse coronavirus translation and is regulated by complexing with Ifit1c. J. Biol. Chem. 295, 17781–17801. https://doi.org/10.1074/jbc.RA120.014695
5. Michaelis, K.A., Norgard, M.A., Levasseur, P.R., Olson, B., Burfeind, K.G., Buenafe, A.C., Zhu, X., Jeng, S., McWeeney, S.K., Marks, D.L., 2019. Persistent Toll-like receptor 7 stimulation induces behavioral and molecular innate immune tolerance. Brain. Behav. Immun. 82, 338–353. https://doi.org/10.1016/j.bbi.2019.09.004
6. Orthgiess, J., Gericke, M., Immig, K., Schulz, A., Hirrlinger, J., Bechmann, I., Eilers, J., 2016. Neurons exhibit *Lyz2* promoter activity in vivo: Implications for using LysM-Cre mice in myeloid cell research: Technical comment. Eur. J. Immunol. 46, 1529–1532. https://doi.org/10.1002/eji.201546108
7. Pidugu, V.K., Pidugu, H.B., Wu, M.-M., Liu, C.-J., Lee, T.-C., 2019. Emerging Functions of Human IFIT Proteins in Cancer. Front. Mol. Biosci. 6, 148. https://doi.org/10.3389/fmolb.2019.00148
8. Sintes, J., Romero, X., De Salort, J., Terhorst, C., Engel, P., 2010. Mouse CD84 is a *pan* -leukocyte cell-surface molecule that modulates LPS-induced cytokine secretion by macrophages. J. Leukoc. Biol. 88, 687–697. https://doi.org/10.1189/jlb.1109756
9. Steffens, N., Beuter-Gunia, C., Kravets, E., Reich, A., Legewie, L., Pfeffer, K., Degrandi, D., 2020. Essential Role of mGBP7 for Survival of Toxoplasma gondii Infection. mBio 11, e02993-19. https://doi.org/10.1128/mBio.02993-19
10. Terenzi, F., White, C., Pal, S., Williams, B.R.G., Sen, G.C., 2007. Tissue-Specific and Inducer-Specific Differential Induction of ISG56 and ISG54 in Mice. J. Virol. 81, 8656–8665. https://doi.org/10.1128/JVI.00322-07
11. Wang, C., Wang, Zeqi, Yao, T., Zhou, J., Wang, Zhaoyang, 2022. The immune-related role of beta-2-microglobulin in melanoma. Front. Oncol. 12, 944722. https://doi.org/10.3389/fonc.2022.944722
